# Sleep Patterns and Affect Dynamics Among College Students During the COVID-19 Pandemic: Intensive Longitudinal Study

**DOI:** 10.2196/33964

**Published:** 2022-08-05

**Authors:** Zahra Avah Mousavi, Jocelyn Lai, Katharine Simon, Alexander P Rivera, Asal Yunusova, Sirui Hu, Sina Labbaf, Salar Jafarlou, Nikil D Dutt, Ramesh C Jain, Amir M Rahmani, Jessica L Borelli

**Affiliations:** 1 Department of Psychological Science University of California, Irvine Irvine, CA United States; 2 Department of Cognitive Sciences University of California, Irvine Irvine, CA United States; 3 Department of Economics University of California, Irvine Irvine, CA United States; 4 Department of Computer Science University of California, Irvine Irvine, CA United States; 5 School of Nursing University of California, Irvine Irvine, CA United States

**Keywords:** sleep, objective sleep outcomes, COVID-19, affect variability, affect dynamics

## Abstract

**Background:**

Sleep disturbance is a transdiagnostic risk factor that is so prevalent among young adults that it is considered a public health epidemic, which has been exacerbated by the COVID-19 pandemic. Sleep may contribute to mental health via affect dynamics. Prior literature on the contribution of sleep to affect is largely based on correlational studies or experiments that do not generalize to the daily lives of young adults. Furthermore, the literature examining the associations between sleep variability and affect dynamics remains scant.

**Objective:**

In an ecologically valid context, using an intensive longitudinal design, we aimed to assess the daily and long-term associations between sleep patterns and affect dynamics among young adults during the COVID-19 pandemic.

**Methods:**

College student participants (N=20; female: 13/20, 65%) wore an Oura ring (Ōura Health Ltd) continuously for 3 months to measure sleep patterns, such as average and variability in total sleep time (TST), wake after sleep onset (WASO), sleep efficiency, and sleep onset latency (SOL), resulting in 1173 unique observations. We administered a daily ecological momentary assessment by using a mobile health app to evaluate positive affect (PA), negative affect (NA), and COVID-19 worry once per day.

**Results:**

Participants with a higher sleep onset latency (*b*=−1.09, SE 0.36; *P*=.006) and TST (*b*=−0.15, SE 0.05; *P*=.008) on the prior day had lower PA on the next day. Further, higher average TST across the 3-month period predicted lower average PA (*b=*−0.36, SE 0.12; *P=*.009). TST variability predicted higher affect variability across all affect domains. Specifically, higher variability in TST was associated higher PA variability (*b=*0.09, SE 0.03; *P=*.007), higher negative affect variability (*b=*0.12, SE 0.05; *P=*.03), and higher COVID-19 worry variability (*b=*0.16, SE 0.07; *P=*.04).

**Conclusions:**

Fluctuating sleep patterns are associated with affect dynamics at the daily and long-term scales. Low PA and affect variability may be potential pathways through which sleep has implications for mental health.

## Introduction

Sleep is a robust and transdiagnostic risk factor for various physical and mental health problems, including mood disorders [1-3]. Indeed, sleep disorders, such as insomnia and circadian misalignment, contribute to the development or recurrence of mood disorders, particularly depression and anxiety [4]. Variability in sleep duration, as measured by day-to-day changes in nightly sleep, is as important a predictor for psychological well-being as the average total amount of sleep [5]. Sleep problems, such as chronic sleep restriction and irregular sleep patterns, are common among college students [6]. For emerging adults who are already at greater risk for psychopathology [7,8], the COVID-19 pandemic has disrupted daily routines [9,10], thereby potentially exacerbating variable sleep patterns and contributing to further insufficient sleep and greater variability in sleep duration. Given the prevalence of sleep disturbances as well as the impact of the pandemic on young adults, it may be important to examine the associations between sleep and affect in young adults during the COVID-19 pandemic.

Insufficient sleep and sleep variability may contribute to physical and mental health via affect dynamics. Positive affect (PA) and negative affect (NA)—broadband indices of emotion—are well-established predictors of well-being [11]. Blunted PA and increased NA are risk factors for and significant predictors of the development and recurrence of mental disorders, such as depression. COVID-19 worry, or concern over SARS-CoV-2 infection, is another relevant affect construct that has arisen during the pandemic [12,13]. COVID-19 worry has been discussed at length by psychologists during the COVID-19 pandemic [12-14] and is a distinct psychological factor that uniquely contributes to general anxiety and persistent pessimism [12]. Affect, however, is dynamic; the trajectory of emotional experiences often fluctuates across time [15]. Beyond average affect, daily affect dynamics, such as affect variability, may contribute to explaining individual differences in psychological functioning. Affect variability—a measure of the extent to which individuals experience frequent PA, NA, and COVID-19 worry fluctuations—is known to play a prominent role in psychopathologies, such as mood disturbances [16,17].

Prior literature has mainly focused on the negative consequences that sleep deprivation has for average PA and NA, finding that sleep duration significantly predicts dampened PA and elevated NA [18-22]. Experimental studies have also confirmed the similar effects of sleep loss on changes in PA and NA [23,24]. Specifically, 1 night of sleep deprivation and/or sleep restriction (ie, 4 hours of sleep), when compared to idealized sleep (ie, the opportunity of sleeping for 9.5-10 hours), decreases PAs such as vigor and increases NAs such as anger [23,24]. Yet, this knowledge is largely based on correlational studies or sleep deprivation experiments that do not generalize to the daily lives of young adults with chronic sleep restriction. Additional studies on the relation between affect and sleep problems in young adults that are conducted in a more ecologically valid context (eg, daily life) may help to elucidate these associations. Furthermore, to date, the literature examining the associations between daily sleep variability and affect dynamics remains scant. Daily sleep variability contributes to psychological well-being [5,25], but more studies that use newer methods to examine objective sleep are needed. Studies with intensive longitudinal designs that are conducted over longer periods of time to assess both subjective and objective sleep outcomes are poised to accomplish these goals. Considering the important contribution of poor sleep to mood disorders, particularly depression and anxiety [4,19], it is important to examine the nuanced characterizations of the consequences that daily sleep disturbances have for affect dynamics. Examining the association between daily sleep and affect will allow for a better understanding of the development of comorbid sleep and mood disorders and how to design prevention programs for at-risk individuals.

The aim of this study was therefore to assess (1) the daily associations between sleep and affect and (2) the long-term associations between sleep patterns (ie, average and variability in total sleep time [TST], wake after sleep onset [WASO], sleep efficiency, sleep onset latency [SOL]) and affect dynamics (ie, mean levels of PA and NA, PA and NA variability, and COVID-19 worry) dynamics among young adults during the COVID-19 pandemic across a 3-month period. Examining these associations at the between- and within-person levels will allow for an improved understanding of the development of comorbid sleep and mood disorders and support the identification of early intervention windows for at-risk individuals. This study was designed to examine sleep among young adults in a 3-month period; however, the period of assessment varied from 1 month to 3 months due to retention. The majority of previous studies have assessed subjective and objective sleep outcomes in a 14-day period [5,25], meaning that our study examined a longer period of time. Further, multilevel modeling is a powerful analytic approach to analyzing intensive longitudinal data with missing values for both between-subject research questions and, especially, within-subject research questions.

## Methods

### Participants

College student participants (N=20; female: 13/20, 65%; age: mean 19.80, SD 1.0 years) were assessed daily across a 3-month period during the 2020 COVID-19 pandemic (June to November), resulting in 1173 unique observations. Participants were eligible if they met the following criteria: participants must be unmarried, be English speakers, be full-time undergraduate students aged between 18 and 22 years, and own a primary Android smartphone that is compatible with the ecological momentary assessment (EMA) phone-based survey apps and study wearable devices.

### Ethics Approval

This study was part of a larger intensive longitudinal study for examining student mental health that included physiological assessments, sleep tracking, and daily emotional and behavioral reports. The procedures of this study were approved by the institutional review board (approval number: 2019-5153) at University of California, Irvine. All individuals provided written informed consent prior to participation.

### Procedure

Herein, we describe the procedures that are relevant to the purposes of our investigation. Participants were first instructed on how to wear the noninvasive device (ie, Oura ring [Ōura Health Ltd]) that continuously assessed sleep, activity, and physiology throughout the day and during sleep [26]. Participants completed daily surveys on affect by using a smartphone app. To maintain high adherence, participants received reminders via text message, email, or phone call if there were more than 2 days of inactivity.

### Measures

#### Sleep

By using the Oura ring (specifications: 2 infrared light-emitting diode heart rate sensors, 2 negative thermal coefficient body temperature sensors, a 3-axis accelerometer, and a gyroscope), TST, WASO, sleep efficiency, and SOL were calculated through the detection and interpretation of physiological measures, including heart rate, heart rate variability, and pulse wave variability amplitude. Previous studies have compared the Oura ring to polysomnography—the gold standard of sleep measurement—and research-grade actigraphy (Philips Respironics). A study by Chee et al [27] shows that, based on an epoch-by-epoch analysis, the Oura ring yields sleep assessments that are comparable to those of actigraphy [27] but underestimates TST when compared to polysomnography. However, other studies, such as one by de Zambotti et al [28], have shown that summary variables for SOL, TST, and WASO are not different between the Oura ring and polysomnography. The validation study by de Zambotti et al [28] was conducted among healthy adolescents and young adults and showed that “the differences for TST and WASO between PSG and Oura are within the ≤ 30 min a-priori-set clinically satisfactory ranges for 87.8% and 85.4% of the sample, respectively.” Their study also showed that the Oura ring is able to categorize sleep, with an accuracy of >81.3%, based on polysomnography-defined TST ranges (eg, <6 hours, 6-7 hour, and >7 hours). However, there are some concerns regarding detecting the stages of sleep (eg, light sleep, deep sleep, and rapid eye movement sleep); therefore, sleep stages were not included in this study.

#### Affect and COVID-19 Worry

As part of the EMA phone-based surveys, participants reported daily PA and NA by using the Positive and Negative Affect Schedule (PANAS) [29] each evening. On the PANAS, 10 positive (eg, inspired) items and 10 negative (eg, nervous) items were rated on a 0 to 100 scale (0=“Very Slightly”; 100=“Extremely”). Total scores for PA and NA were calculated by using the average across each 10-item subscale (PA: mean 45.27, SD 20.22; *α*=.85; NA: mean 21.79, SD 12.28; *α*=.91). As a separate item (“How worried were you about contracting COVID today?”), participants reported their COVID-19 worry on a 0 to 100 scale (mean 17.41, SD 19.15).

#### Sleep and Affect Variability

To determine the variability of each sleep and affect variable, we first created a series of successive differences by calculating the difference between 2 successive observations within the same subject (eg, night 2 − night 1; night 3 − night 2; etc). Next, these values were squared. We used the square successive differences to compute a mean square successive difference score. Finally, we calculated the root mean square successive difference score for each participant. The root mean square successive difference is considered an index of variability that is similar to the intraindividual variance of a series of observations but is more sensitive to fluctuations across successive observations [30].

### Data Analysis

We first examined variables for normality and heteroscedasticity. To examine the association between sleep (TST, sleep efficiency, and SOL) and affect variables (PA, NA, and COVID-19 worry), we first conducted multilevel models by using the restricted maximum likelihood approach. This approach improves estimates of variance components and fixed effect SE estimates in smaller samples by separating the estimates of the fixed effects from the variance components [31]. We predicted PA, NA, and COVID-19 worry as a function of the fixed effects of time and between- and within-subject sleep variables while controlling for previous-day affect. Next, we examined the association between sleep and affect variables by using multiple regression models. Previous studies have found that sex is linked with sleep outcomes [32-34]. Therefore, sex was included as a covariate in these regression models. Finally, we conducted hierarchical linear regressions to examine whether sleep variability variables contribute to affect dynamics above and beyond the average sleep variables.

## Results

### Descriptive Statistics

A total of 20 college students (female: 13/20, 65%) completed this study, providing 1623 (mean 43.49, SD 25.51 days/person) nights of usable Oura ring sleep data. Completion rates for EMA studies were high (83%).

Table S1 in Multimedia Appendix 1 provides descriptive statistics and the bivariate correlations between the key variables. The averages for participants’ TST and TST variability were 6.84 and 1.8 hours, respectively. Participants also experienced an average of 65.04 and 12.01 minutes of WASO and SOL, respectively, with a sleep efficiency of 86.52% across the study. Further, on average, participants reported low levels of NA (mean 21.79), moderate levels of PA (mean 45.27), and low levels of COVID-19 worry (mean 17.41) across the study. We assessed the bivariate correlations between the COVID-19 worry mean and PA and NA means (between-person level) and found no significant correlations between these constructs. Specifically, the correlation between the COVID-19 worry mean and PA mean was −0.19 (*P*=.43), and the correlation between the COVID-19 worry mean and NA mean was 0.43 (*P*=.06).

### Daily Sleep and Daily Affect

The multilevel models of the relation between sleep and affect revealed that participants with a higher SOL (*b*=−1.09, SE 0.36; *P*=.006) and TST on the prior day (*b*=−0.15, SE 0.05; *P*=.008) had lower PA on the next day, while controlling for previous-day PA. No within-subject differences were observed in predictions for next-day PA. No associations between daily sleep and NA and between daily sleep and COVID-19 worry were found. Table S2 in Multimedia Appendix 2 shows more details.

### Main Effects of Sleep on Average Affect

The regression model for predicting PA from average TST accounted for 34% of the variance in average PA (adjusted *R*^2^=0.26; *F*_2,17_=4.24; *P*=.03). Specifically, higher TST was associated with lower PA (*b=*−0.36, SE 0.12; *P=*.009). Other sleep variables were not associated with average PA. Sleep was not associated with average NA or COVID-19 worry. Table S3 in Multimedia Appendix 3 shows more details.

### Main Effects of Sleep on Affect Variability

#### Sleep Averages and Affect Variability

SOL and sleep efficiency predicted COVID-19 worry variability. The multiple regression model for predicting COVID-19 worry variability from the average SOL and sex accounted for 38% of the variance in COVID-19 worry variability (adjusted *R*^2^=0.31; *F*_2,17_=5.29; *P*=.02), and the model for predicting COVID-19 worry variability from average sleep efficiency and sex accounted for 38% of the variance in COVID-19 worry variability (adjusted *R*^2^=0.31; *F*_2,17_=5.24; *P*=.02). Specifically, higher average SOL predicted higher COVID-19 worry variability (*b=*1.87, SE 0.89; *P=*.05), and higher sleep efficiency predicted lower COVID-19 worry variability (*b=*−2.02, SE 0.97; *P=*.05). Table S4 in Multimedia Appendix 4 shows more details.

#### Sleep Variability and Affect Variability

The multiple regression models for predicting affect variability from TST variability, while controlling for sex, accounted for 36% of the variance in PA variability (adjusted *R*^2^=0.29; *F*_2,17_=4.82; *P*=.02), 34% of the variance in NA variability (adjusted *R*^2^=0.27; *F*_2,17_=4.44; *P*=.03), and 40% of the variance in COVID-19 worry variability (adjusted *R*^2^=0.33; *F*_2,17_=5.70; *P*=.01). Specifically, higher variability in TST was associated higher PA variability (*b*=0.09, SE 0.03; *P=*.007), higher NA variability (*b*=0.12, SE 0.05; *P=*.03), and higher COVID-19 worry variability (*b*=0.16, SE 0.07; *P*=.04). Table S4 in Multimedia Appendix 4 shows more details.

The hierarchical regression models showed that TST variability predicted PA variability above and beyond the average TST (adjusted *R*^2^=0.26; *F*_3,16_=3.20; *P*=.05). However, the models for predicting NA variability and COVID-19 worry variability were not statistically significant after adding average TST to the models (adjusted *R*^2^=0.22; *F*_3,16_=2.82; *P*=.07 and adjusted *R*^2^=0.22; *F*_3,16_=2.82; *P*=.07, respectively).

## Discussion

### Principal Findings

Sleep patterns across the daily and long-term scales were associated with daily and average affect and affect variability, which were assessed over a 3-month period. These findings are consistent with those of prior work suggesting that poor sleep confers a heightened risk for affective disturbances that are prevalent in mood disorders, such as depression [4]. The link between sleep variability and affect variability may provide a window into how such patterns develop over time.

Individuals with longer sleep times on the previous night experienced lower PA on the next day. Similarly, individuals with longer average TSTs over the study period reported lower PA. Previous studies however have suggested a positive association between sleep duration and greater PA (eg, a study by Galambos et al [25]). During the COVID-19 pandemic, young adults’ lives have changed dramatically [35], and it is possible that they have been sleeping for longer than the recommended hours; therefore, there may be a curvilinear association between TST and PA. Future studies may benefit from examining this curvilinear association in a larger sample. Notably, consistent with prior studies [36], sleep did not predict NA. Low PA may be a potential pathway through which sleep duration has implications for mental health. Low PA is both a significant predictor of the onset of depression and a characteristic of depressive disorders. When low PA is directly treated, symptoms of anxiety and depression, as well as other disorders with anhedonic features, are known to improve [37]. Thus, improving daily sleep may be particularly important for preventing PA decline, thereby potentially interrupting the pathogenesis of illness states, such as mood disorders.

TST variability predicted higher affect variability across all affect domains (ie, PA, NA, COVID-19 worry); thus, it may be a proximal predictor of mood disturbances. Affect variability is prevalent in various mood-related disorders, such as depression, bipolar disorder, and anxiety disorders [38]. Our findings have important implications. First, interventions that support sleep stability may indirectly reduce affect variability and therefore prevent clinically significant mood disturbances. For example, in bipolar disorder, variable sleep and affect during euthymia predict worse long-term outcomes, including episodic relapses [39]. Second, our findings suggest that the stabilization of affect may be an early marker or predictor of the efficacy of transdiagnostic sleep interventions that target mood disorders with anhedonic features [39]. Future research may benefit from experimentally investigating the effect of regulating sleep time on affect dynamics in clinical populations.

Our findings also provide specific relevance to the COVID-19 context. Individuals with a higher SOL experienced higher COVID-19 worry variability, and individuals with a higher sleep efficiency reported lower COVID-19 worry variability. The specificity of the associations between SOL and sleep efficiency, and COVID-19 worry variability (ie, not NA in general) may suggest that the association between sleep and arousal-related affect is unique. Sleep disturbances may contribute to the lower capacity to adaptively overcome stress and therefore may be associated with higher stress-related sleep reactivity and cognitive presleep hyperarousal [40]. Future research may benefit from experimentally investigating the association between sleep and specific subtypes of affect.

### Limitations

Our findings carry some limitations. First, whether sleep causally predicts affect remains unclear. Daily affect may also predict multiple sleep indices [36]. The bidirectional link between sleep and affect may result in a cyclical pattern of sleep disturbance impacting affect, which in turn may contribute to greater sleep disturbances. Second, the small sample size of this study was not diverse in terms of age, which limits the generalizability of our findings to other age groups. However, the peak prevalence of affect variability is among adolescents and young adults (ie, individuals aged 16-24 years) and gradually declines with age [38]. Considering the small sample size of this study, future studies may benefit from examining the association between sleep patterns and affect dynamics in a larger sample.

### Conclusion

Fluctuating sleep patterns are associated with affect dynamics, such as average PA and affect variability, across all affect domains (ie, PA, NA, COVID-19 worry) at the daily and long-term scales. Low PA and affect variability may be potential pathways through which sleep has implications for mental health. Interventions that target sleep stability may indirectly reduce affect variability and therefore prevent mood disorders. The stabilization of affect may be an early marker or predictor of the efficacy of transdiagnostic sleep interventions that target mood disorders with anhedonic features.

## Appendix

Multimedia Appendix 1 Means, SDs, and correlations between sleep and affect variables.

Multimedia Appendix 2 Adjusted estimates for predicting average affect from objective sleep, gender, and age.

Multimedia Appendix 3 Adjusted estimates for predicting affect variability from objective sleep, gender, and age.

Multimedia Appendix 4 Unstandardized coefficient estimates in models for predicting daily sleep by daily affect.

## Abbreviations

EMA: ecological momentary assessment
NA: negative affect
PA: positive affect
PANAS: Positive and Negative Affect Schedule
SOL: sleep onset latency
TST: total sleep time
WASO: wake after sleep onset

## References

[1] Baglioni C, Battagliese G, Feige B, Spiegelhalder K, Nissen C, Voderholzer U, Lombardo C, Riemann D (2011). Insomnia as a predictor of depression: a meta-analytic evaluation of longitudinal epidemiological studies. J Affect Disord.

[2] Harvey AG (2008). Insomnia, psychiatric disorders, and the transdiagnostic perspective. Curr Dir Psychol Sci.

[3] Irwin MR (2015). Why sleep is important for health: a psychoneuroimmunology perspective. Annu Rev Psychol.

[4] Murray JM, Sletten TL, Magee M, Gordon C, Lovato N, Bartlett DJ, Kennaway DJ, Lack LC, Grunstein RR, Lockley SW, Rajaratnam SMW, Delayed Sleep on Melatonin (DelSoM) Study Group (2017). Prevalence of circadian misalignment and its association with depressive symptoms in delayed sleep phase disorder. Sleep.

[5] Fuligni AJ, Hardway C (2006). Daily variation in adolescents' sleep, activities, and psychological well-being. J Res Adolesc.

[6] Lund HG, Reider BD, Whiting AB, Prichard JR (2010). Sleep patterns and predictors of disturbed sleep in a large population of college students. J Adolesc Health.

[7] Leebens PK, Williamson ED (2017). Developmental psychopathology: Risk and resilience in the transition to young adulthood. Child Adolesc Psychiatr Clin N Am.

[8] Weitzman ER (2004). Poor mental health, depression, and associations with alcohol consumption, harm, and abuse in a national sample of young adults in college. J Nerv Ment Dis.

[9] Lai J, Rahmani A, Yunusova A, Rivera AP, Labbaf S, Hu S, Dutt N, Jain R, Borelli JL (2021). Using multimodal assessments to capture personalized contexts of college student well-being in 2020: Case study. JMIR Form Res.

[10] Savage MJ, James R, Magistro D, Donaldson J, Healy LC, Nevill M, Hennis PJ (2020). Mental health and movement behaviour during the COVID-19 pandemic in UK university students: Prospective cohort study. Ment Health Phys Act.

[11] Diener E, Scollon CN, Lucas RE, Diener E (2009). The evolving concept of subjective well-being: The multifaceted nature of happiness. Assessing Well-Being: The Collected Works of Ed Diener.

[12] Jungmann SM, Witthöft M (2020). Health anxiety, cyberchondria, and coping in the current COVID-19 pandemic: Which factors are related to coronavirus anxiety?. J Anxiety Disord.

[13] Limcaoco RSG, Mateos EM, Fernández JM, Roncero C Anxiety, worry and perceived stress in the world due to the COVID-19 pandemic, March 2020. Preliminary results. medRxiv.

[14] Muñoz-Navarro R, Malonda E, Llorca-Mestre A, Cano-Vindel A, Fernández-Berrocal P (2021). Worry about COVID-19 contagion and general anxiety: Moderation and mediation effects of cognitive emotion regulation. J Psychiatr Res.

[15] Ebner-Priemer UW, Eid M, Kleindienst N, Stabenow S, Trull TJ (2009). Analytic strategies for understanding affective (in)stability and other dynamic processes in psychopathology. J Abnorm Psychol.

[16] Houben M, Van Den Noortgate W, Kuppens P (2015). The relation between short-term emotion dynamics and psychological well-being: A meta-analysis. Psychol Bull.

[17] Trull TJ, Lane SP, Koval P, Ebner-Priemer UW (2015). Affective dynamics in psychopathology. Emot Rev.

[18] Dahl RE, Lewin DS (2002). Pathways to adolescent health sleep regulation and behavior. J Adolesc Health.

[19] Short MA, Booth SA, Omar O, Ostlundh L, Arora T (2020). The relationship between sleep duration and mood in adolescents: A systematic review and meta-analysis. Sleep Med Rev.

[20] De Valck E, Cluydts R (2001). Slow-release caffeine as a countermeasure to driver sleepiness induced by partial sleep deprivation. J Sleep Res.

[21] Yoo SS, Gujar N, Hu P, Jolesz FA, Walker MP (2007). The human emotional brain without sleep--a prefrontal amygdala disconnect. Curr Biol.

[22] Zohar D, Tzischinsky O, Epstein R, Lavie P (2005). The effects of sleep loss on medical residents' emotional reactions to work events: a cognitive-energy model. Sleep.

[23] Short MA, Louca M (2015). Sleep deprivation leads to mood deficits in healthy adolescents. Sleep Med.

[24] Reddy R, Palmer CA, Jackson C, Farris SG, Alfano CA (2017). Impact of sleep restriction versus idealized sleep on emotional experience, reactivity and regulation in healthy adolescents. J Sleep Res.

[25] Galambos NL, Dalton AL, Maggs JL (2009). Losing sleep over it: Daily variation in sleep quantity and quality in Canadian students' first semester of university. J Res Adolesc.

[26] Mehrabadi MA, Azimi I, Sarhaddi F, Axelin A, Niela-Vilén H, Myllyntausta S, Stenholm S, Dutt N, Liljeberg P, Rahmani AM (2020). Sleep tracking of a commercially available smart ring and smartwatch against medical-grade actigraphy in everyday settings: Instrument validation study. JMIR Mhealth Uhealth.

[27] Chee NIYN, Ghorbani S, Golkashani HA, Leong RLF, Ong JL, Chee MWL (2021). Multi-night validation of a sleep tracking ring in adolescents compared with a research actigraph and polysomnography. Nat Sci Sleep.

[28] de Zambotti M, Rosas L, Colrain IM, Baker FC (2019). The sleep of the ring: Comparison of the ŌURA sleep tracker against polysomnography. Behav Sleep Med.

[29] Watson D, Clark LA, Tellegen A (1988). Development and validation of brief measures of positive and negative affect: the PANAS scales. J Pers Soc Psychol.

[30] Jahng S, Wood PK, Trull TJ (2008). Analysis of affective instability in ecological momentary assessment: Indices using successive difference and group comparison via multilevel modeling. Psychol Methods.

[31] McNeish D (2017). Small sample methods for multilevel modeling: A colloquial elucidation of REML and the Kenward-Roger correction. Multivariate Behav Res.

[32] Goel N, Kim H, Lao RP (2005). Gender differences in polysomnographic sleep in young healthy sleepers. Chronobiol Int.

[33] Krishnan V, Collop NA (2006). Gender differences in sleep disorders. Curr Opin Pulm Med.

[34] Zhang B, Wing YK (2006). Sex differences in insomnia: a meta-analysis. Sleep.

[35] Glowacz F, Schmits E (2020). Psychological distress during the COVID-19 lockdown: The young adults most at risk. Psychiatry Res.

[36] Kalmbach DA, Pillai V, Roth T, Drake CL (2014). The interplay between daily affect and sleep: a 2-week study of young women. J Sleep Res.

[37] Craske MG, Meuret AE, Ritz T, Treanor M, Dour HJ (2016). Treatment for anhedonia: A neuroscience driven approach. Depress Anxiety.

[38] Marwaha S, Parsons N, Flanagan S, Broome M (2013). The prevalence and clinical associations of mood instability in adults living in England: results from the Adult Psychiatric Morbidity Survey 2007. Psychiatry Res.

[39] Broome MR, Saunders KEA, Harrison PJ, Marwaha S (2015). Mood instability: significance, definition and measurement. Br J Psychiatry.

[40] Palagini L, Petri E, Novi M, Caruso D, Moretto U, Riemann D (2018). Adult insecure attachment plays a role in hyperarousal and emotion dysregulation in insomnia disorder. Psychiatry Res.

