# Supplementary material for: Sleep Patterns and Affect Dynamics Among College Students During the COVID-19 Pandemic: Intensive Longitudinal Study
Source: JMIR Form Res. 2022 Aug 5;6(8):e33964. doi: 10.2196/33964 (PMC9359303; doi:10.2196/33964)
Supplement: Multimedia Appendix 1 [file formative_v6i8e33964_app1.docx]

| Table S1  *Means, standard deviations and correlations between sleep and affect variables* | | | | | | | | | | | | | | | | | | | | | | | | | | | | | | | |
| --- | --- | --- | --- | --- | --- | --- | --- | --- | --- | --- | --- | --- | --- | --- | --- | --- | --- | --- | --- | --- | --- | --- | --- | --- | --- | --- | --- | --- | --- | --- | --- |
| Correlations | | | | | | | | | | | | | | | | | | | | | | | | | | | | | | | |
|  | M(SD) | | 1 | | 2 | | 3 | | 4 | | 5 | | 6 | | 7 | | 8 | | 9 | | 10 | | 11 | | 12 | | 13 | | 14 | | 15 |
| 1. Gender |  | 1 | |  | |  | |  | |  | |  | |  | |  | |  | |  | |  | |  | |  | |  | |  | |
| Sleep |  |  | |  | |  | |  | |  | |  | |  | |  | |  | |  | |  | |  | |  | |  | |  | |
| 1. Total sleep time mean | 410.24 (33.62) | -.18 | | 1 | |  | |  | |  | |  | |  | |  | |  | |  | |  | |  | |  | |  | |  | |
| 1. WASO mean | 65.04 (15.89) | .59** | | -.19 | | 1 | |  | |  | |  | |  | |  | |  | |  | |  | |  | |  | |  | |  | |
| 1. Sleep efficiency mean | 86.52 (3.13) | -.60** | | .47* | | -.95** | | 1 | |  | |  | |  | |  | |  | |  | |  | |  | |  | |  | |  | |
| 1. SOL mean | 12.01 (2.99) | -.43^†^ | | .35 | | -.11 | | .28 | | 1 | |  | |  | |  | |  | |  | |  | |  | |  | |  | |  | |
| 1. Total sleep time variability | 108.90 (33.82) | .22 | | -.32 | | .18 | | -.25 | | .07 | | 1 | |  | |  | |  | |  | |  | |  | |  | |  | |  | |
| 1. WASO variability | 43.87 (17.25) | .02 | | .01 | | .27 | | -.12 | | .32 | | .15 | | 1 | |  | |  | |  | |  | |  | |  | |  | |  | |
| 1. Sleep efficiency variability | 6.99 (1.82) | .003 | | -.01 | | .26 | | -.17 | | .36 | | -.01 | | .80** | | 1 | |  | |  | |  | |  | |  | |  | |  | |
| 1. SOL variability | 14.61 (6.57) | -.40^†^ | | .36 | | .07 | | .08 | | .79** | | -.18 | | .38^†^ | | .58** | | 1 | |  | |  | |  | |  | |  | |  | |
| Affect |  |  | |  | |  | |  | |  | |  | |  | |  | |  | |  | |  | |  | |  | |  | |  | |
| 1. PA mean | 45.27 (20.22) | .04 | | -.58** | | .03 | | -.23 | | -.43^†^ | | .13 | | -.22 | | -.29 | | -.38^†^ | | 1 | |  | |  | |  | |  | |  | |
| 1. NA mean | 21.79 (12.28) | -.53* | | .24 | | -.28 | | .32 | | .27 | | -.13 | | -.11 | | -.09 | | .29 | | -.001 | | 1 | |  | |  | |  | |  | |
| 1. COVID-worry mean | 17.41 (19.15) | -.48* | | .04 | | -.08 | | .12 | | .19 | | .22 | | .10 | | .15 | | .19 | | -.19 | | .43^†^ | | 1 | |  | |  | |  | |
| 1. PA variability | 12.37 (5.12) | -.11 | | -.26 | | .04 | | -.07 | | .33 | | .55* | | .20 | | .004 | | -.01 | | .08 | | .31 | | .23 | | 1 | |  | |  | |
| 1. NA variability | 13.94 (8.98) | -.37 | | -.10 | | -.13 | | .10 | | .45* | | .36 | | .01 | | -.18 | | .14 | | .17 | | .46* | | .33 | | .74** | | 1 | |  | |
| 1. COVID-worry variability | 16.26 (12.67) | -.47* | | -.13 | | .003 | | -.03 | | .56** | | .31 | | .13 | | .15 | | .41^†^ | | .09 | | .41 | | .56** | | .48* | | .70** | | 1 | |
| *Note:* † *p*<.10.  ** p*<.05.  *** p*<.01. | | | | | | | | | | | | | | | | | | | | | | | | | | | | | | | |
