# Supplementary material for: Sleep Patterns and Affect Dynamics Among College Students During the COVID-19 Pandemic: Intensive Longitudinal Study
Source: JMIR Form Res. 2022 Aug 5;6(8):e33964. doi: 10.2196/33964 (PMC9359303; doi:10.2196/33964)
Supplement: Multimedia Appendix 2 [file formative_v6i8e33964_app2.docx]

| Table S2  *Unstandardized coefficient estimates in models predicting daily sleep by daily affect* | | | | | | | | | | | | | | |
| --- | --- | --- | --- | --- | --- | --- | --- | --- | --- | --- | --- | --- | --- | --- |
|  | Positive affect | | | | | Negative affect | | | | | COVID-worry | | | |
|  | *b(SE)* | *p* | | 95% CI | | *b(SE)* | *p* | | 95% CI | | *b(SE)* | *p* | 95% CI | |
|  |  | |  | *LL* | *UL* |  | |  | *LL* | *UL* |  |  | *LL* | *UL* |
| **Model 1** |  | |  |  |  |  | |  |  |  |  |  |  |  |
| Intercept | 16.77 (1.60) | | <.001 | 13.58 | 19.96 | 7.98 (1.39) | | <.001 | 5.14 | 10.83 | 11.54 (2.55) | <.001 | 6.24 | 16.84 |
| Day | .02 (.01) | | .09 | -.003 | .04 | .02 (.01) | | .06 | -.001 | .04 | -.03 (.02) | .12 | -.06 | .01 |
| SOL between-subject | -1.09 (.36) | | .006 | -1.84 | -.34 | .38 (.41) | | .37 | -.49 | 1.25 | 1.13 (.79) | .17 | -.54 | 2.81 |
| SOL within-subject | -.02 (.99) | | .98 | -2.12 | 2.07 | .01 (.03) | | .58 | -.04 | .07 | -.05 (.05) | .36 | -.16 | .06 |
| PA last day | .62 (.02) | | <.001 | .57 | .66 |  | |  |  |  |  |  |  |  |
| NA last day |  | |  |  |  | .57 (.03) | | <.001 | .52 | .62 |  |  |  |  |
| COVID-worry last day |  | |  |  |  |  | |  |  |  | .43 (.03) | <.001 | .37 | .49 |
| **Model 2** |  | |  |  |  |  | |  |  |  |  |  |  |  |
| Intercept | 18.65 (2.03) | | <.001 | 14.52 | 22.78 | 8.36 (1.44) | | <.001 | 5.39 | 11.33 | 11.82 (2.72) | <.001 | 6.13 | 17.52 |
| Day | .01 (.01) | | .16 | -.01 | .03 | .02 (.01) | | .08 | -.002 | .04 | -.02 (.02) | .15 | -.06 | .01 |
| TST between-subject | -.15 (.05) | | .008 | -.25 | -.04 | .03 (.04) | | .38 | -.04 | .11 | .05 (.07) | .49 | -.11 | .21 |
| TST within-subject | -.008 (.005) | | .13 | -.02 | .002 | -.007 (.005) | | .11 | -.02 | .001 | -.005 (.01) | .54 | -.02 | .01 |
| PA last day | .56 (.02) | | <.001 | .51 | .61 |  | |  |  |  |  |  |  |  |
| NA last day |  | |  |  |  | .56 (.03) | | <.001 | .51 | .62 |  |  |  |  |
| COVID-worry last day |  | |  |  |  |  | |  |  |  | .42 (.03) | <.001 | .36 | .48 |
| **Model 3** |  | |  |  |  |  | |  |  |  |  |  |  |  |
| Intercept | 19.40 (2.37) | | <.001 | 14.49 | 24.31 | 8.57 (1.46) | | <.001 | 5.55 | 11.59 | 11.77 (2.74) | <.001 | 6.02 | 17.52 |
| Day | .01 (.01) | | .16 | -.005 | .03 | .02 (.01) | | .06 | -.001 | .04 | -.02 (.02) | .18 | -.05 | .01 |
| SE between-subject | -.61 (.63) | | .35 | -1.95 | .73 | .53 (.42) | | .22 | -.35 | 1.41 | .50 (.82) | .55 | -1.28 | 2.27 |
| SE within-subject | .005 (.06) | | .94 | -.12 | .13 | -.03 (.07) | | .65 | -.16 | .10 | -.01 (.10) | .90 | -.20 | .18 |
| PA last day | .55 (.02) | | <.001 | .50 | .61 |  | |  |  |  |  |  |  |  |
| NA last day |  | |  |  |  | .55 (.03) | | <.001 | .49 | .60 |  |  |  |  |
| COVID-worry last day |  | |  |  |  |  | |  |  |  | .41 (.03) | <.001 | .35 | .47 |
