# Supplementary material for: Sleep Patterns and Affect Dynamics Among College Students During the COVID-19 Pandemic: Intensive Longitudinal Study
Source: JMIR Form Res. 2022 Aug 5;6(8):e33964. doi: 10.2196/33964 (PMC9359303; doi:10.2196/33964)
Supplement: Multimedia Appendix 3 [file formative_v6i8e33964_app3.docx]

| Table S3  Adjusted estimates predicting average affect from objective sleep, gender, and age | | | | | | | | | | | | | | | | | | | | |
| --- | --- | --- | --- | --- | --- | --- | --- | --- | --- | --- | --- | --- | --- | --- | --- | --- | --- | --- | --- | --- |
|  | PA mean | | | | | | | NA mean | | | | | | | COVID- worry mean | | | | | |
|  | *b(SE)* | *p* | | 95% CI | | | *b(SE)* | | *p* | | | 95% CI | | *b(SE)* | | *p* | | | 95% CI | |
| **Model 1** |  | |  | | *LL* | *UL* |  | | |  | *LL* | | *UL* |  | | |  | *LL* | | *UL* |
| Total sleep time mean | -.36 (.12) | | .009 | | -.64 | -.10 | .05 (.07) | | | .48 | -.10 | | .17 | -.02 (.12) | | | .84 | -.26 | | .23 |
| Gender | -2.34 (6.75) | | .73 | | -15.05 | 17.52 | -10.35 (4.22) | | | .02 | -23.82 | | -2.13 | -15.63 (6.91) | | | .04 | -29.17 | | -7.70 |
| *R*^2^ | .34 | |  | |  |  | .30 | | |  |  | |  | .23 | | |  |  | |  |
| F | 4.24 | | .03 | |  |  | 3.69 | | | .05 |  | |  | 2.58 | | | .10 |  | |  |
| **Model 2** |  | |  | |  |  |  | | |  |  | |  |  | | |  |  | |  |
| WASO mean | .01 (.38) | | .98 | | -.83 | .81 | .05 (.20) | | | .81 | -.49 | | .51 | .38 (.30) | | | .23 | -.33 | | 1.05 |
| Gender | 1.11 (10.19) | | .91 | | -22.55 | 30.80 | -11.65 (5.24) | | | .04 | -24.56 | | -2.21 | -21.37 (8.11) | | | .02 | -36.95 | | -8.16 |
| *R*^2^ | .001 | |  | |  |  | .28 | | |  |  | |  | .29 | | |  |  | |  |
| F | .01 | | .99 | |  |  | 3.38 | | | .06 |  | |  | 3.55 | | | .05 |  | |  |
| **Model 3** |  | |  | |  |  |  | | |  |  | |  |  | | |  |  | |  |
| Sleep efficiency mean | -2.18 (1.89) | | .27 | | -5.80 | 1.88 | -.03 (1.01) | | | .97 | -.03 | | .97 | -1.66 (1.58) | | | .31 | -5.37 | | 1.45 |
| Gender | -5.66 (9.91) | | .57 | | -26.10 | 26.35 | -10.99 (5.3) | | | .05 | -2.07 | | .05 | -20.65 (8.29) | | | .02 | -38.45 | | -8.39 |
| *R*^2^ | .07 | |  | |  |  | .28 | | |  |  | |  | .28 | | |  |  | |  |
| F | .67 | | .52 | |  |  | 3.34 | | | .06 |  | |  | 3.26 | | | .06 |  | |  |
| **Model 4** |  | |  | |  |  |  | | |  |  | |  |  | | |  |  | |  |
| SOL mean | -3.40 (1.61) | | .05 | | -5.95 | -.18 | .23 (.93) | | | .81 | -2.01 | | 2.03 | .77 (1.50) | | | .61 | -2.51 | | 3.85 |
| Gender | -6.03 (8.07) | | .46 | | -20.30 | 18.89 | -10.34 (-1.62) | | | .04 | -30.00 | | -1.86 | -13.74 (7.47) | | | .08 | -31.84 | | -4.87 |
| *R*^2^ | .21 | |  | |  |  | .28 | | |  |  | |  | .24 | | |  |  | |  |
| F | 2.24 | | .14 | |  |  | 3.38 | | | .06 |  | |  | 2.72 | | | .09 |  | |  |
| **Model 5** |  | |  | |  |  |  | | |  |  | |  |  | | |  |  | |  |
| Total sleep time variability | .07 (.15) | | .62 | | -.38 | .35 | -.004 (.08) | | | .95 | -.20 | | .10 | .19 (.11) | | | .11 | -.01 | | .45 |
| Gender | .30 (8.34) | | .97 | | -16.76 | 26.35 | -10.84 (4.33) | | | .02 | -23.44 | | -2.00 | -17.82 (6.46) | | | .01 | -29.62 | | -7.48 |
| *R*^2^ | .02 | |  | |  |  | .28 | | |  |  | |  | .34 | | |  |  | |  |
| F | .14 | | .87 | |  |  | 3.34 | | | .06 |  | |  | 4.43 | | | .03 |  | |  |
| **Model 6** |  | |  | |  |  |  | | |  |  | |  |  | | |  |  | |  |
| WASO variability | -.26 (.28) | | .36 | | -1.02 | .31 | -.07 (.14) | | | .62 | -.28 | | .40 | .13 (.23) | | | .60 | -.25 | | 1.42 |
| Gender | 1.39 (7.99) | | .86 | | -14.09 | 23.30 | -10.85 (4.19) | | | .02 | -25.25 | | -3.72 | -15.45 (6.75) | | | .03 | -28.36 | | -7.06 |
| *R*^2^ | .05 | |  | |  |  | .29 | | |  |  | |  | .24 | | |  |  | |  |
| F | .45 | | .64 | |  |  | 3.52 | | | .05 |  | |  | 2.74 | | | .09 |  | |  |
| **Model 7** |  | |  | |  |  |  | | |  |  | |  |  | | |  |  | |  |
| Sleep efficiency variability | -3.26 (2.57) | | .22 | | -8.79 | .29 | -.58 (1.38) | | | .68 | -2.66 | | 2.26 | 1.64 (2.20) | | | .47 | -2.59 | | 8.61 |
| Gender | 1.27 (7.83) | | .87 | | -13.41 | 25.94 | -10.89 (4.20) | | | .02 | -24.34 | | -3.91 | -15.39 (6.70) | | | .03 | -28.20 | | -6.57 |
| *R*^2^ | .09 | |  | |  |  | .29 | | |  |  | |  | .25 | | |  |  | |  |
| F | .81 | | .46 | |  |  | 3.45 | | | .05 |  | |  | 2.91 | | | .08 |  | |  |
| **Model 8** |  | |  | |  |  |  | | |  |  | |  |  | | |  |  | |  |
| SOL variability | -1.33 (.75) | | .09 | | -2.74 | -.14 | .16 (.42) | | | .70 | -.80 | | .97 | .003 (.68) | | | .99 | -1.32 | | 1/61 |
| Gender | -4.62 (8.20) | | .58 | | -21.42 | 19.34 | -10.18 (4.58) | | | .04 | -27.48 | | -1.75 | -15/37 (7.43) | | | .05 | -30.36 | | -5.00 |
| *R*^2^ | .16 | |  | |  |  | .29 | | |  |  | |  | .23 | | |  |  | |  |
| F | 1.61 | | .23 | |  |  | 3.44 | | | .056 |  | |  | 2.55 | | | .11 |  | |  |
