# Supplementary material for: Sleep Patterns and Affect Dynamics Among College Students During the COVID-19 Pandemic: Intensive Longitudinal Study
Source: JMIR Form Res. 2022 Aug 5;6(8):e33964. doi: 10.2196/33964 (PMC9359303; doi:10.2196/33964)
Supplement: Multimedia Appendix 4 [file formative_v6i8e33964_app4.docx]

| Table S4  Adjusted estimates predicting affect variability from objective sleep, gender, and age | | | | | | | | | | | | | | | | | | | | |
| --- | --- | --- | --- | --- | --- | --- | --- | --- | --- | --- | --- | --- | --- | --- | --- | --- | --- | --- | --- | --- |
|  | PA variability | | | | | | | NA variability | | | | | | | COVID- worry variability | | | | | |
|  | *b(SE)* | *p* | | 95% CI | | | *b(SE)* | | *p* | | | 95% CI | | *b(SE)* | | *p* | | 95% CI | | |
| **Model 1** |  | |  | | *LL* | *UL* |  | | |  | *LL* | | *UL* |  | | |  | | *LL* | *UL* |
| Total sleep time mean | -.04 (.04) | | .24 | | -.13 | .03 | -.05 (.06) | | | .45 | -.20 | | .09 | -.08 (.08) | | | .32 | | -.26 | .07 |
| Gender | -1.42 (2.01) | | .49 | | -8.93 | 2.07 | -6.09 (3.37) | | | .09 | -16.18 | | -1.20 | -10.85 (4.46) | | | .03 | | -20.84 | -3.18 |
| *R*^2^ | .09 | |  | |  |  | .17 | | |  |  | |  | .27 | | |  | |  |  |
| F | .84 | | .44 | |  |  | 1.74 | | | .20 |  | |  | 3.14 | | | .07 | |  |  |
| **Model 2** |  | |  | |  |  |  | | |  |  | |  |  | | |  | |  |  |
| WASO mean | .05 (.10) | | .58 | | 0.1 | .36 | .08 (.16) | | | .63 | -.19 | | .44 | .35 (.19) | | | .09 | | .035 | .72 |
| Gender | -1.83 (2.54) | | .48 | | -9.39 | 2.38 | -6.83 (4.17) | | | .12 | -17.55 | | -.66 | -15.56 (5.16) | | | .008 | | -25.55 | -8.35 |
| *R*^2^ | .03 | |  | |  |  | .15 | | |  |  | |  | .35 | | |  | |  |  |
| F | .27 | | .76 | |  |  | 1.52 | | | .25 |  | |  | 4.56 | | | .03 | |  |  |
| **Model 3** |  | |  | |  |  |  | | |  |  | |  |  | | |  | |  |  |
| Sleep efficiency mean | -.37 (.49) | | .46 | | -1.77 | .50 | -.56 (.80) | | | .49 | -2.36 | | .93 | -2.02 (.97) | | | .05 | | -3.89 | -.25 |
| Gender | -2.15 (2.55) | | .41 | | -10.77 | 2.25 | -7.39 (4.18) | | | .09 | -18.91 | | -.63 | -16.41 (5.08) | | | .005 | | -28.15 | -8.54 |
| *R*^2^ | .04 | |  | |  |  | .16 | | |  |  | |  | .38 | | |  | |  |  |
| F | .40 | | .67 | |  |  | 1.67 | | | .22 |  | |  | 5.24 | | | .02 | |  |  |
| **Model 4** |  | |  | |  |  |  | | |  |  | |  |  | | |  | |  |  |
| SOL mean | .37 (.45) | | .41 | | -.56 | 1.17 | 1.07 (.70) | | | .14 | -.12 | | 2.23 | 1.87 (.89) | | | .05 | |  |  |
| Gender | -.18 (2.23) | | .94 | | -7.35 | 2.95 | -3.33 (3.50) | | | .35 | -10.48 | | .45 | -6.03 (4.46) | | | .19 | |  |  |
| *R*^2^ | .05 | |  | |  |  | .24 | | |  |  | |  | .38 | | |  | |  |  |
| F | .47 | | .63 | |  |  | 2.74 | | | .09 |  | |  | 5.29 | | | .02 | |  |  |
| **Model 5** |  | |  | |  |  |  | | |  |  | |  |  | | |  | |  |  |
| Total sleep time variability | .09 (.03) | | .007 | | .02 | .13 | .12 (.05) | | | .03 | -.10 | | .22 | .16 (.07) | | | .04 | | -.03 | .28 |
| Gender | -2.13 (1.70) | | .23 | | -6.74 | .77 | -7.16 (3.03) | | | .03 | -13.52 | | -.18 | -12.06 (4.08) | | | .009 | | -17.93 | -2.73 |
| *R*^2^ | .36 | |  | |  |  | .34 | | |  |  | |  | .40 | | |  | |  |  |
| F | 4.82 | | .02 | |  |  | 4.44 | | | .03 |  | |  | 5.70 | | | .01 | |  |  |
| **Model 6** |  | |  | |  |  |  | | |  |  | |  |  | | |  | |  |  |
| WASO variability | .06 (.07) | | .40 | | -.02 | .36 | .01 (.12) | | | .94 | -.15 | | .56 | .10 (.15) | | | .53 | | -.12 | .99 |
| Gender | -1.02 (2.02) | | .62 | | -6.71 | 2.02 | -5.62 (3.38) | | | .11 | -14.65 | | -1.23 | -10.08 (4.47) | | | .04 | | -19.25 | -3.02 |
| *R*^2^ | .05 | |  | |  |  | .14 | | |  |  | |  | .24 | | |  | |  |  |
| F | .49 | | .62 | |  |  | 1.39 | | | .28 |  | |  | 2.72 | | | .09 | |  |  |
| **Model 7** |  | |  | |  |  |  | | |  |  | |  |  | | |  | |  |  |
| Sleep efficiency variability | .01 (.68) | | .99 | | -1.36 | 1.09 | -.87 (1.09) | | | .44 | -3.11 | | 1.61 | 1.04 (1.46) | | | .49 | | -2.44 | 4.82 |
| Gender | -.98 (2.06) | | .64 | | -6.84 | 2.17 | -5.61 (3.31) | | | .11 | -13.88 | | -1.04 | -10.03 (4.46) | | | .04 | | -17.38 | -3.36 |
| *R*^2^ | .01 | |  | |  |  | .17 | | |  |  | |  | .25 | | |  | |  |  |
| F | .11 | | .89 | |  |  | 1.76 | | | .20 |  | |  | 2.78 | | | .09 | |  |  |
| **Model 8** |  | |  | |  |  |  | | |  |  | |  |  | | |  | |  |  |
| SOL variability | -.05 (.20) | | .79 | | -.46 | .21 | -.02 (.33) | | | .95 | -.72 | | .43 | .50 (.43) | | | .26 | | -.40 | 1.41 |
| Gender | -1.22 (2.24) | | .59 | | -7.80 | 2.44 | -5.72 (3.68) | | | .14 | -15.28 | | -.66 | -7.83 (4.75) | | | .12 | | -16.94 | -.35 |
| *R*^2^ | .02 | |  | |  |  | .14 | | |  |  | |  | .28 | | |  | |  |  |
| F | .15 | | .86 | |  |  | 1.39 | | | .28 |  | |  | 3.31 | | | .06 | |  |  |
